# The Behavioral Intervention Technology Model and Intervention Mapping: The Best of Both Worlds

**DOI:** 10.2196/jmir.3620

**Published:** 2014-08-05

**Authors:** Rik Crutzen

**Affiliations:** ^1^CAPHRIDepartment of Health PromotionMaastricht UniversityMaastrichtNetherlands

**Keywords:** mHealth, eHealth, behavioral intervention technology, intervention mapping

Mohr and colleagues recently presented an important paper describing an integrated conceptual and technological framework for eHealth and mHealth interventions, using existing models as their point of departure [1]. They mentioned that the focus of existing *psychological* models on clinical outcomes instead of behavior is one of two limitations. However, behavior is a central element in both of the provided examples—the theory of planned behavior and the social cognitive theory. The second limitation was that “psychological models do not include critically important factors that can guide the design and specifications for a BIT (behavioral intervention technology)” [1]. The idea of describing a BIT model that supports the translation of intervention components into features is accepted with open arms.

The BIT model was developed based on a review of three *design* models proposed by Ritterband [2], Fogg [3], and Oinas-Kukkonen [4], and addresses the limitations of these existing design models. Although the authors of the paper acknowledged that the review was not exhaustive, consideration of the intervention mapping (IM) protocol [5,6] would have added value to this viewpoint paper. The limitations of the existing design models are also addressed within IM, and there is overlap between the BIT model and IM, but each has their own unique contributions as well [7].

IM is a protocol for developing theory- and evidence-based interventions. IM describes the development process in six steps: (1) needs assessment, (2) specifying performance objectives and change objectives, (3) selecting theory-based intervention methods and practical applications, (4) designing and organizing the intervention, (5) specifying adoption and implementation plans, and (6) generating an evaluation plan. The protocol guides developers through each of these steps by means of specific tasks. I will focus on steps (2)-(4), as these steps are related to the limitations highlighted in the viewpoint paper by Mohr et al.

In step 2 of IM, the required actions (ie, performance objectives) for the behavioral outcomes have to be specified. Performance objectives are specific sub-behaviors of the health promoting behavior. For example, when you like to promote condom use (health promoting behavior), you want people to obtain condoms in order to use them. This is in line with the BIT component *Aims*. In the BIT model, attention is being paid to usage aims. Although use can be considered as adoption and implementation at the individual level [8], it is good to think ahead and consider use as a behavior with its own determinants [9]. Therefore, in IM, each performance objective is crossed with its determinants, resulting in the formulation of change objectives. These are specific goals of an intervention—to change the determinants of sub-behaviors.

In step 3 of IM, methods and applications are selected to influence the determinants in the desired direction. This step circumvents the limitations of existing design models in that “the Ritterband model does not articulate how technological components might be mapped onto more specific (and promixal) intervention goals” and the Fogg Behavior Model “does not purport to guide applications focused on changing attitudes or cognitions” [1].

A theoretical method is a general technique or process for influencing changes in the determinants, whereas an application is a specific technique for the practical use of theoretical methods in the context of the intervention. This is reflected in the BIT components *Behavior change strategies* and *Elements,* but parameters for use are not mentioned in the BIT model. The parameters for use are conditions that need to be met for a practical application to accurately reflect the theoretical method. If these parameters are lost in translation from method to application, then the method may not be used correctly and its effectiveness might be undermined. For example, feedback as a method works well if the feedback is personalized, follows behavior in time, and is specific [10]. This is different from the method of providing information about others’ approval, which can only work optimally if positive expectations are available in the environment [11].

In step 4 of IM, the methods and applications are organized in a program plan. This gets round the limitation of the Oinas-Kukkonen model that “does not discuss how individual intervention elements may be varied or integrated into a larger treatment program” [1]. IM has been used to develop eHealth or mHealth interventions [12,13] but is applicable to other intervention types. The BIT model provides tools that are very useful and more specific to the context of eHealth and mHealth interventions (ie, the BIT-Tech aspect of the model).

In sum, both the BIT model and IM address the limitations of existing models. Despite considerable overlap, they each have a unique contribution. Whereas IM stresses the importance of parameters for use, the BIT model focuses on the technical instantiation. The BIT model and IM are complementary, each with their own qualities. For example, when using IM, the BIT-Tech aspect of the BIT model is deemed useful in step 4. On the other hand, when using the BIT model, intervention developers should take the parameters for use from IM into account and report this in the research protocol [14]. In my opinion, the unique contributions of both should be valued during the intervention development process. 
